# Clinical Utility and Validation of the Acoustic Voice Quality and Acoustic Breathiness Indexes for Voice Disorder Assessment in English Speakers

**DOI:** 10.3390/jcm12247679

**Published:** 2023-12-14

**Authors:** Adrián Castillo-Allendes, Juliana Codino, Lady Catherine Cantor-Cutiva, Charles J. Nudelman, Adam D. Rubin, Ben Barsties v. Latoszek, Eric J. Hunter

**Affiliations:** 1 Department of Communicative Sciences and Disorders, Michigan State University, East Lansing, MI 48824, USA; casti208@msu.edu (A.C.-A.);; 2 Department of Communication Sciences and Disorders, University of Iowa, Iowa City, IA 52242, USA; 3 Lakeshore Professional Voice Center, Lakeshore Ear, Nose & Throat Center, St. Clair Shores, MI 48081, USA; 4 Department of Speech and Hearing Science, University of Illinois Urbana-Champaign, Champaign, IL 61820, USA; 5 Speech-Language Pathology, SRH University of Applied Health Sciences, 40210 Düsseldorf, Germany

**Keywords:** AVQI, voice quality, breathiness, voice disorders, dysphonia, voice assessment, auditory-perceptual assessment, acoustic voice

## Abstract

Background: While several acoustic voice metrics are available for clinical voice assessment, there remains a significant need for reliable and ecologically valid tools. The Acoustic Voice Quality Index version 03.01 (AVQI-3) and Acoustic Breathiness Index (ABI) hold potential due to their comprehensive assessment approach, incorporating diverse voice aspects. However, these tools still need to be validated in English-speaking populations. Methods: This study assessed the discriminatory accuracy and validity of AVQI-3 and ABI in 197 participants, including 148 with voice disorders. Voice samples were collected, followed by AVQI-3 and ABI calculations. Additionally, auditory-perceptual assessments were conducted by a panel of speech-language pathologists. Results: AVQI-3 and ABI effectively identified disordered voice quality, evidenced by high accuracy (AUCs: 0.84, 0.89), sensitivity, and specificity (thresholds: AVQI-3 = 1.17, ABI = 2.35). Strong positive correlations were observed with subjective voice quality assessments (rs = 0.72, rs = 0.77, *p* < 0.001). Conclusions: The study highlights AVQI-3 and ABI as promising instruments for clinically assessing voice disorders in U.S. English speakers, underscoring their utility in clinical practice and voice research.

## 1. Introduction

Voice quality assessment is pivotal in evaluating voice disorders and dysphonia, contributing to accurate diagnosis and effective treatment planning [1,2,3]. While auditory-perceptual judgment has been widely used as the gold standard for evaluating voice quality, its subjective nature and potential limitations in terms of validity and reliability have prompted the development of objective assessment methods [4,5]. Acoustic analysis of the voice signal has emerged as a promising approach, providing a reliable and objective tool for quantifying voice disorders in both research and clinical settings [6,7]. However, the use of multiple acoustical parameters during vocal assessment has limitations which can be addressed by indexes that combine multiple voice parameters to aid in the quantification of voice production quality. Among various indexes utilized in voice assessment, such as the Cepstral Spectral Index of Dysphonia (CSID) and the Dysphonia Severity Index (DSI) [8,9], this study primarily focuses on the Acoustic Voice Quality Index (AVQI) and the Acoustic Breathiness Index (ABI). These later two multiparametric models aim to quantify overall voice quality and assess breathiness severity, respectively.

The Acoustic Voice Quality Index (AVQI) is a multiparametric model that presents an objective and standardized approach to assess overall voice quality. It encompasses acoustic markers obtained from continuous speech and sustained vowel samples, enabling an integral evaluation of the overall voice quality [10]. By incorporating various parameters from both the temporal and frequency domains, the AVQI generates a single score that reflects the overall voice quality via acoustic analysis rather than a single score through auditory-perceptual judgments [10]. While there have been several versions of the AVQI, the latest, AVQI v03.01 (AVQI-3), addresses previous limitations by incorporating proportional adjustments. These enhancements provide an equalized emphasis on different speech parts, thereby improving its validity and adaptability, especially in clinical settings. The adjustments are particularly effective in enhancing the AVQI-3’s ability to differentiate between various voice qualities and dysphonia severities, resulting in improved discriminatory precision [10,11].

On the other hand, the ABI is a tool that specifically assesses vocal breathiness [12]. Vocal breathiness is a perceptual characteristic of voice quality related to the extent of air leakage through the glottis. It is often associated with conditions such as phono-traumatic masses, acute laryngitis, vocal fold paralysis or paresis, vocal fold bowing, and posterior glottic diastasis [13,14]. The ABI estimates the degree of breathiness using nine acoustic parameters. As with the AVQI, it analyzes concatenated speech and voice samples. The ABI has been validated in many languages. Studies demonstrate its strong correlations with perceptual breathiness scores, and it has high discriminatory accuracy and sensitivity to therapy-related voice quality [12].

Using indexes based on multiple metrics, such as the AVQI-3 and ABI, is an important direction to provide a usable assessment of voice disorder severity for tracking treatment outcomes. However, previous studies have shown that these indexes may be sensitive to linguistic context and must be assessed in various languages for ecological validity [10,12,15]. Previous work has successfully assessed AVQI-3 and ABI in a range of languages, including Dutch, Spanish, Japanese, Korean, Brazilian Portuguese, and German, among others, with favorable results in terms of its robustness, inter-language phonetic differences, and sensitivity to changes in dysphonia severity [10,12,16].

Therefore, this paper reports work to assess the AVQI-3 and ABI specifically for the English language, providing insights into their performance in a distinct linguistic environment. By establishing the robustness, accuracy, and reliability of indexes such as the AVQI-3 and ABI in assessing English speakers’ overall voice quality and breathiness, this study will contribute to the growing body of literature evaluating the value of this multi-acoustical parameter approach. Further, in assessing the clinical utility of these two specific indexes, the results may allow for the further diagnostic and therapeutic capabilities of these acoustic indexes as valuable tools for clinicians and researchers in the field of voice disorders.

## 2. Materials and Methods

This prospective cross-sectional study is designed to assess the ecological validity of the AVQI-3 and ABI for English. The methodology closely aligns with previous investigations focused on validating AVQI-3 and ABI across various languages. 

### 2.1. Participants

Participant voice data came from two separate locations, recordings of patients with voice disorders from the Lakeshore Professional Voice Center (St. Clair Shores, MI, USA) and non-clinic-seeking adults recruited by The Voice Biomechanics and Acoustics Laboratory at Michigan State University. Table 1 presents the demographic information of the adults providing the recordings, including gender (as a biological factor) and age, and voice diagnosis. The voice disorders (*n* = 148) sample represented a range of dysphonia etiologies. The non-clinic-seeking sample (*n* = 49) was recruited from the general population to age match the voice disorder group; only those reporting neither vocal complaints nor voice disorders were included in the “normal voice” group. As a confirmation metric, all normal voice participants completed the voice handicap index (VHI) with results within the normality ranges. Voice recordings from both groups were collected under similar conditions, using the same microphone and audio interface, and with similar background noise levels.

### 2.2. Voice Samples

Recordings included two voicing tasks: (1) a sustained vowel [a:] performed at a comfortable pitch and loudness, and (2) an oral reading, using a habitual voice, of the first three sentences of the phonetically balanced text “the Rainbow Passage [17]”. The collection protocol allowed for the text to be read before the voice recording to reduce common variability due to lack of familiarity, misreading, or mispronunciation. Audio recordings were obtained using a head-mounted microphone (Shure BETA 54 super-cardioid condenser positioned 5 cm from the speaker’s mouth, manufactured by Shure Incorporated, Niles, IL, USA) connected to a Focusrite Scarlett Solo (3rd gen) audio interface (Focusrite Audio Engineering Ltd., High Wycombe, Buckinghamshire, UK) using an In-Line Preamp adapter (RPM627, Shure Incorporated, Niles, IL, USA). All recordings (wav, 44.1 kHz, 16-bit) were collected in a quiet environment with ambient noise levels below 35 dB. To ensure adherence to recommended norms, the signal-to-noise ratio (SNR) was measured for each recording using the method by Deliyski et al. [2,18]. The minimum SNR of the voice recordings was required to exceed 30 dB as a post-hoc control of the environmental noise level. 

### 2.3. Validation Process

The validation process, based on previous reports, comprises two phases. The first phase that focuses on assessing ecological validity included determining a standardized US (United States) English syllable number (SSN) corresponding to 3 s of continuous speech (CS) (Rainbow passage). In the second phase, the following four steps were completed: (1) assessment of auditory-perceptual judgment reliability; (2) evaluation of differences of AVQI-3 values between auditory-perceptually identified healthy and unhealthy voices; (3) correlation assessment between AVQI-3 with auditory-perceptual judgments; and (4) definition of the optimal cut-off value and discriminatory accuracy of the AVQI-3.

#### 2.3.1. Phase One

##### Standardized Syllable Number for the Continuous Speech (CS) Part

This phase consisted of two parts, which provided the appropriate cutoff point for the number of syllables in the CS portion, allowing for an accurate calculation of the AVQI-3 and ABI measures. First, voiceless CS segments (vCS) were extracted from the CS using the Praat software (version 6.3.06) [19], using the extraction Praat script developed by Maryn et al. [20] A customized cutoff point was then established by hand-marking the original text, corresponding to the extracted first 3 s obtained in the previous step. The duration of each hand-marked segment was verified, and the extraction Praat-script by Maryn et al. [20] was rerun on the customized segment, allowing for a tolerant margin of ±0.1 s between the segment and the extracted portion. Second, the total syllable count across all voice samples was obtained, and the range and mean of syllables were calculated. Moreover, 95% confidence intervals (CI) were established. Subsequently, the upper and lower boundary values of syllables within the unextracted voice sample were identified using the CI.

#### 2.3.2. Phase Two

##### Auditory-Perceptual Assessment

**Figure 1:**
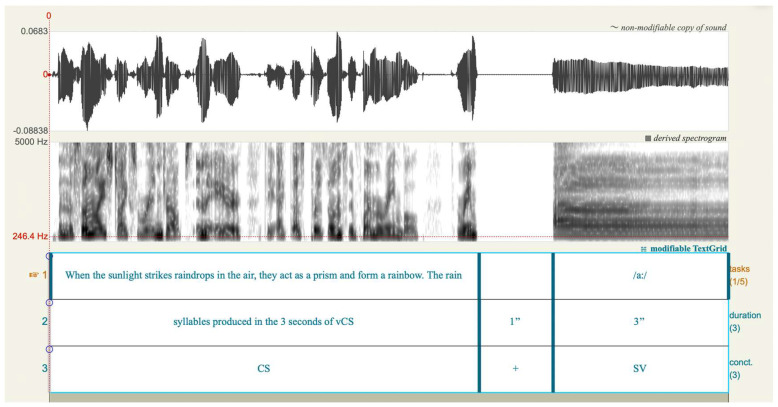
Praat visualization of the concatenated audio sample used for AVQI-3 and ABI validation. The display consists of a waveform (**top**), a derived spectrogram (**middle**), and a modifiable TextGrid (**bottom**). Tier 1 on the TextGrid captures the first 22 syllables from the Rainbow Passage, succeeded by a 1 s silence and then a 3 s segment of the sustained vowel /a:/. Tier 2 indicates the duration of each task, providing clarity on the temporal length of individual audio segments. Tier 3 offers a categorical breakdown: “CS” corresponds to continuous speech derived from the Rainbow Passage, while “SV” designates the sustained vowel.

The first part of the second phase involved auditory-perceptual assessment using the GRBAS scale, a widely recognized tool for evaluating voice quality [21,22]. An expert panel of six speech-language pathologists, each with more than two years of experience in assessing voice quality and working in the field of voice and voice disorders, participated in the assessment. The panel was blinded to the identity and diagnosis of the voice samples, and they then individually rated the overall voice quality of each concatenated piece, see Figure 1. While the GRBAS scale assesses multiple perceptual aspects of voice (i.e., grade or overall voice quality, G; roughness, R; breathiness, B; asthenia, A; and strain, S), only G and B parameters will be employed in this investigation for the validation of the AVQI-3 and ABI, respectively. To determine inter-rater reliability, a randomly selected subset comprising 20% of voice samples was duplicated to identify the consistency of ratings [23]. Following the auditory-perceptual assessments, the six raters’ intra-rater and inter-rater reliabilities were assessed for each judgment procedure, with any instances of weak inter- or intra-rater reliability considered grounds for rater exclusion.

##### Acoustic Measures

In parallel with the auditory-perceptual evaluation, acoustic measures were performed on the voiced segments of CS, and a 3 s segment of the sustained vowel [a:]. The acoustic analysis included the calculation of six parameters for the Acoustic Voice Quality Index (AVQI): smoothed cepstral peak prominence (CPPs), harmonics-to-noise ratio (HNR), shimmer local (Shim), shimmer local dB (ShdB), general slope of the spectrum (Slope), and tilt of the regression line through the spectrum (Tilt). The calculation of these parameters for the AVQI are based on the equation proposed by Barsties and Maryn [12]:

AVQIV3 = [4.152 − (0.177 × CPPs) − (0.006 × HNR) − (0.037 × Shim) + (0.941 × ShdB) + (0.01 × Slope) + (0.093 × Tilt)] × 2.8902

The parameters of the ABI encompass not only the previously mentioned parameters (i.e., CPPs, Shim, and ShdB), but also include jitter local (Jit), glottal-to-noise excitation ratio (GNEmax-4500 Hz), high-frequency noise at 6000 Hz (Hno-6000 Hz), the harmonics-to-noise ratio of Dejonckere (HNR-D) [24], differences in the amplitudes of the first and second harmonics of the spectrum (H1-H2), and period standard deviation (PSD). These parameters have undergone thorough investigation in various prior studies. To calculate the ABI equations according to Barsties v. Latoszek [12], the following factors are considered:

ABI = [5.0447740915 − (0.172 × CPPs) − (0.193 × Jit) − (1.283 × GNEmax-4500 Hz) − (0.396 × Hno-6000 Hz) + (0.01 × HNR-D) + (0.017 × H1-H2) + (1.473 × ShdB) − (0.088 × Shim) − (68.295 × PSD)] × 2.9257400394

To facilitate the analysis of the AVQI-3 and ABI indexes, VOXplot version 2.0.0 software (Lingphon, Straubenhardt, Germany) was employed. This software incorporates established Praat software algorithms and uses the same equation, ensuring consistent and reliable results.

### 2.4. Statistical Analysis

Statistical analyses were executed using SPSS software (IBM Corp. Released 2021. IBM SPSS Statistics for Windows, Version 28.0. Armonk, NY, USA: IBM Corp.) as well as RStudio software (RStudio Team, v. 4.3.1, R Core Team, Vienna, Austria).

First, the standardized duration of the CS sample was assessed. The standardized syllable number was established by rounding the hand-marked syllable Field’s lower 95% confidence interval (CI) boundary [20]. Subsequently, a comparison was performed between the standardized selection of syllables and the hand-marked selection of syllables within the CS segment, encompassing an evaluation of time and the AVQI-3 and ABI outcomes. This comparative analysis was executed using the Wilcoxon signed-rank test, with statistical significance attributed to findings reaching a *p*-value of ≤0.01.

To evaluate both the agreement of intra-rater and inter-rater reliability, statistical analyses were conducted using the Cohen’s kappa coefficient (Ck) for G and B parameters, as well as Fleiss’ kappa (Fk), which extends the Cohen’s kappa for situations involving multiple judges. The interpretation of kappa values is based on Landis and Koch [25]. This reliability assessment was conducted employing RStudio software. Furthermore, significant changes (i.e., considered statistically significant at *p* ≤ 0.01) in kappa values were tested using bootstrapping with 1000 replications based on a script by Vanbelle [26].

Additionally, to determine the criterion-related concurrent validities of the AVQI-3 and ABI, the Spearman rank-order correlation coefficient (r_s_) and the coefficient of determination (r_s_^2^) were employed. This involved investigating the associations between perceptual average judgments of overall voice quality (G parameter) and the AVQI-3 and between perceptual average judgments of breathiness (B parameter) and the ABI. 

To determine the discriminatory accuracy of the AVQI-3 and ABI, a receiver operating characteristic (ROC) curve analysis was conducted. Following the recommendation of Barsties and Maryn [20], voices were classified as healthy voices (normophonic) (G_mean_ < 0.5) or dysphonic (G_mean_ ≥ 0.5). The discriminatory prowess was quantified by calculating the area under the curve (AUC) and interpreted according to Swets’ guidelines [27]. 

Optimal thresholds for the AVQI-3 and ABI were determined using the Youden Index (sensitivity + specificity − 1), designed to identify the most fitting cut-off score accounting for both sensitivity and specificity [28]. Likelihood ratios (LR) were computed to gauge the applicability of the AVQI-3 and ABI thresholds for clinical decision-making. Accounting for sensitivity and specificity, LR is less affected by disparities in sample sizes between participants with voice disorders and healthy voice speakers. The likelihood ratio for a positive result (LR+) estimates the chance that an individual is dysphonic when the test result is positive, while the likelihood ratio for a negative result (LR−) estimates the chance that an individual has a healthy voice when the test result is negative. Generally, a test’s diagnostic accuracy is considered high when LR+ is ≥10 and LR− is ≤0.1 [29].

## 3. Results

### 3.1. Standardized Syllable Number (SSN) for the Continuous Speech (CS) Part

In determining the appropriate syllable number for achieving a 3 s duration in the CS segment, a spectrum ranging from 11 to 47 syllables was observed. The lower 95%-CI limit, twenty-two (22) syllables, was selected as a candidate for SSN. The comparison between the hand-marked syllable count and the standardized 22-syllable selection for time and ABI yielded no statistically significant differences (*p* = 0.935 and *p* = 0.115, respectively). On the other hand, a statistically significant difference was found when contrasting AVQI-3 outcomes between the hand-marked syllable count and the standardized 22-syllable selection exhibited significant differences (*p* = 0.002) (see Table 2). Notably, the correlation between the two AVQI measurements registered was 0.996 (*p* < 0.01). Subsequently, for later analyses, the CS segment for English was tailored under the SSN count of twenty-two, as exemplified by the following phrase: “When the sunlight strikes raindrops in the air, they act as a prism and form a rainbow. The rain”.

### 3.2. Auditory-Perceptual Assessment: Reliability

The assessment of intra-rater reliability among the six judges yielded Ck values ranging from 0.56 to 0.80 (mean = 0.66) for the G parameter and 0.46 to 0.68 (mean = 0.56) for the B parameter, with no significant differences in the Ck values among the raters for G and B parameters (*p* = 0.039, and *p* = 0.685, respectively). This finding reflects moderate to substantial agreement for both dimensions of voice quality in intra-rater reliability. The Fk value was determined as 0.23 for the G parameter and 0.21 for the B parameter among the judges, indicating a fair level of inter-rater reliability in assessing perceptual overall voice quality and breathiness. The bootstrapping analyses indicated a significant improvement in both Fk values (*p* < 0.01) if a rater from the initial panel was excluded. However, with a rater panel of five remaining, no increase to minimal moderate Fk was obtained in the interpretation guideline by Landis and Koch [24]. Therefore, the original number of six judges was retained for further analysis.

### 3.3. Concurrent Validity

**Figure 2:**
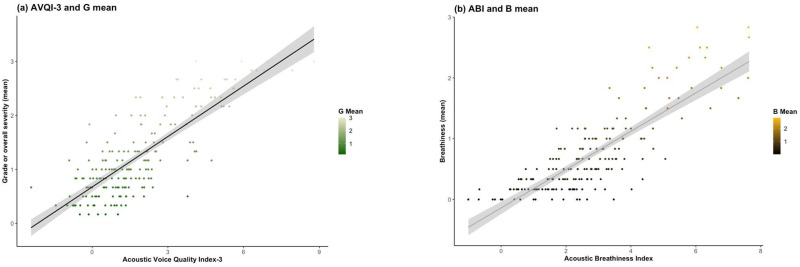
Association between AVQI-3 and G Score (**a**) and between ABI and B score (**b**). Statistically significant concurrent validity linking the AVQI-3 and ABI scores with auditory perceptual evaluations of the G and B parameters (r_s_ = 0.72, *p* < 0.001; r_s_ = 0.77, *p* < 0.001).

The Spearman rank-order correlation coefficient and coefficient of determination unveiled a robust positive correlation between perceptual ratings and AVQI-3 as well as ABI (Figure 2). The findings highlight a statistically significant concurrent validity linking the AVQI-3 and ABI scores with auditory perceptual evaluations of the G and B parameters (r_s_ = 0.72, *p* < 0.001; r_s_ = 0.77, *p* < 0.001). The coefficient of determination, manifested through r_s_^2^ values of 0.52 and 0.59, elucidated the degree to which 52% and 59% of the variability in G_mean_ and B_mean_, respectively, could be elucidated by the predictive efficacy inherent in AVQI-3 and ABI.

### 3.4. Discriminatory Accuracy

The ROC curve of AVQI-3 is shown in Figure 2a. An area under the curve (AUC) of 0.84 indicates a moderately good discriminative ability of AVQI-3 in distinguishing between the voice clinic group and the matched controls. The optimal threshold for AVQI-3, yielding the highest Youden Index value, was 1.17. This value effectively distinguishes normal and hoarse voices within the context of the US English language. This threshold achieves good discriminatory performance with a sensitivity of 62% and specificity of 95%. At this threshold, the likelihood ratio (LR) computation yielded an LR+ of 12.46, showcasing the test’s robust capacity to identify positive cases. Correspondingly, an LR− of 0.40 demonstrates a moderate yet substantial ability to exclude negative cases, underlining a notable discriminatory accuracy range for AVQI-3.

**Figure 3:**
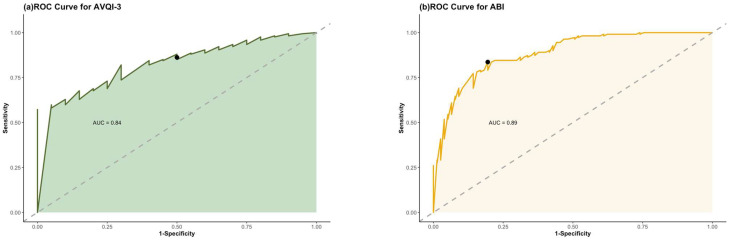
ROC Curves for AVQI-3 and ABI in English. (**a**) AVQI-3 ROC curve (AUC = 0.84): Good discrimination between healthy and hoarse voices; optimal threshold 1.17 (black point) sensitivity = 62%, specificity = 95%). (**b**) ABI ROC curve (AUC = 0.89): High discrimination between breathy and nonbreathy voices; optimal threshold 2.35 (sensitivity = 84%, specificity = 81%). The dashed lines represent the line of no discrimination, indicating predictive ability equal to chance.

Figure 3b illustrates that the AUC for ABI is 0.89, reflecting its strong ability to distinguish between breathy and nonbreathy voices. The optimal threshold for ABI, set at 2.35 based on the Youden Index, results in a closely aligned sensitivity of 84% and specificity of 81%. This demonstrates ABI’s effective and nearly symmetrical balance in discriminating between the two voice types. Additionally, the likelihood ratio analysis at this threshold yields an LR+ of 4.29, emphasizing the test’s capacity to identify positive cases within the clinical group effectively, further reinforcing its utility; an LR− of 0.2 highlights the test’s commendable ability to accurately exclude negative cases in the healthy controls, further endorsing ABI’s strong ability for confidently discriminating between the clinical group and healthy controls regarding the presence or absence of the condition.

## 4. Discussion

This study has comprehensively evaluated the discriminatory accuracy and validity of the Acoustic Voice Quality Index (AVQI-3) and the Acoustic Breathiness Index (ABI) within the context of US English speakers, yielding valuable insights. These findings align with prior research of the AVQI-3 and ABI in languages such as Dutch, Spanish, French, Japanese, Korean, Brazilian Portuguese, Italian, and German [10,12,16]. However, this study represents the first report of AVQI-3 and ABI for US English speakers, marking a significant contribution to the field.

An optimal Standardized Syllable Number of 22 for achieving a 3 s duration in continuous speech (CS) was identified, harmonizing effectively with manually marked syllable counts. This streamlines practical application and enhances the representation of voice quality characteristics in US English.

The study demonstrated fair inter-rater reliability among the judges assessing overall voice quality (G) and breathiness (B), as indicated by Fleiss’ kappa values of 0.23 and 0.21, respectively, and no statistically significant differences. Concurrent validity analyses further substantiated these findings, revealing significant correlations between perceptual ratings and AVQI-3 and ABI scores, affirming their validity regarding auditory-perceptual evaluations of the G and B parameters.

In assessing discrimination accuracy, receiver operating characteristic (ROC) curve assessments confirm AVQI-3’s ability to effectively differentiate between healthy and disordered voices, as indicated by an AUC of 0.84. The threshold set at 1.17 intentionally favors specificity (95%) over sensitivity (62%), reflecting a deliberate choice in its diagnostic application. This preference for high specificity to reduce false negatives is particularly noteworthy, considering the 33% difference from its sensitivity rate. This is crucial for avoiding unnecessary actions and reducing false positives’ psychological and vocational impact, particularly in professional and occupational voice users [30]. It also ensures cost-efficient healthcare resource allocation and optimal treatment for those genuinely affected [31,32]. While prioritizing specificity, this approach acknowledges the sensitivity’s limitations, suggesting a complementary multi-step diagnostic process for a more comprehensive assessment [1,2,33].

Concerning prior studies, our findings agree with a recent comprehensive meta-analysis conducted by Batthyany et al. [10]. That study encompasses diverse languages and incorporates five articles that employ AVQI versions as voice assessment tools for English speakers. While these articles did not have the primary objective of validating AVQI for English, they provide valuable insights within a broader framework.

Reynolds et al. (2012) utilized AVQI version 1, observing notable specificity (92%) and sensitivity 82%) in differentiating healthy from disordered voices [15]. However, it is worth noting that their evaluation encompassed a smaller sample size (107 total voice samples) than the present study, with a focus on the pediatric population, potentially influencing the generalizability of their findings.

Maryn et al. (2014) reported high specificity and sensitivity (90–95%) with AVQI version 1. However, their study featured a limited number of participants (*n* = 50) and voice quality raters (*n* = 3), hampering the generalizability of their reliability and validity results. These issues are contextualized in the wider research overview provided by Batthyany et al. [10].

In Rubin et al.’s work (2018), AVQI version 2 was employed to assess changes in pitch strength following medialization laryngoplasty [34]. While their research highlighted the potential utility of AVQI for specific clinical applications, such as the assessment of vocal fold paralysis, it was conducted with a small number of voice samples (*n* = 22), and sensitivity and specificity values were not reported, as they were not aligned with the research objectives. Consequently, the applicability of their findings to a broader range of voice disorders may be limited due to the constrained sample size.

Lee et al. (2018) conducted a study employing AVQI versions 2 and 3 to compare AVQI’s performance with another tool for acoustic voice outcomes, reporting reliability values (ranging from 0.87 to 0.96) similar to our study [35]. However, they did not provide AUC, sensitivity, or specificity values for AVQI-3. It is pertinent to highlight that their study did not aim to validate AVQI-3 and did not incorporate an ecological approach.

These prior studies collectively contribute to understanding AVQI’s capabilities and limitations in various contexts, underscoring the significance of this current research in formally validating AVQI-3 and enhancing its ecological relevance.

Regarding ABI, our validation of this index within English-speaking populations resonates with the meta-analysis by Barsties v. Latoszek et al. (2021) [12]. It is crucial to recognize that this meta-analysis did not consider English-speaking populations. This was not due to an exclusion criterion but rather because of the limited availability of formal validation studies in this linguistic domain. This scarcity highlights the significance of our research, which thoroughly fills this void by formally validating ABI within an English-speaking context. Moreover, the same meta-analysis incorporated data from various linguistic backgrounds, highlighting ABI’s robust discrimination accuracy. The pooled sensitivity of 0.84 and specificity of 0.92, along with an impressive area under the curve (AUC) of 0.94 in their summary receiver operating characteristic curve, collectively affirmed ABI’s proficiency in distinguishing between healthy and voice-disordered individuals.

Our results reinforced the broader implications of the meta-analysis and unveiled ABI’s heightened discriminatory power compared to our AVQI-3 results. ABI achieved an AUC of 0.89, signifying its enhanced efficacy in distinguishing individuals with breathy and nonbreathy voices. We established an optimal threshold of 2.35, striking a careful balance between sensitivity (84%) and specificity (81%). Additionally, likelihood ratio analysis underscored ABI’s discrimination prowess, yielding an LR+ of 4.29, indicating its accuracy in identifying positive cases (breathy voices), and an LR− of 0.2, signifying its capability to exclude negative cases (nonbreathy voices) reliably. In clinical terms, this means that when ABI suggests the presence of a breathy voice, it is likely to be accurate, and when it indicates the absence of a breathy voice, it is also dependable. This strengthens ABI’s role as a valuable tool for assessing breathy voice quality in clinical practice, aiding in precise diagnoses.

### 4.1. Considerations and Future Directions

Several factors merit consideration in the interpretation of our findings. While we endeavored to enhance the discriminative capacity and detection precision of AVQI-3 and ABI, there is room for further refinement. 

We must also consider the inter-rater reliabilities for G and B scores in interpreting the results. The Fleiss kappa values of 0.23 and 0.21, falling into the ‘Fair agreement’ category as per Landis and Koch [25], indicate the potential for improved consistency in future studies. However, these figures should be viewed within the broader context of the study’s overall robust findings and substantial intra-rater agreement. While these values may suggest room for methodological refinement, it is essential to emphasize that they do not significantly undermine the overall validity of our findings. Instead, they highlight an area for potential improvement in future research.

Notably, our study’s composition of the healthy voice group primarily featured young US women who frequently employed vocal fry [36]—a voice register often utilized volitionally for sociolinguistic purposes [37,38]. Although a natural speech element for many individuals, vocal fry occasionally registers as disordered voice quality for some raters [39]. Consequently, in our study, some individuals with healthy voices may have been erroneously identified as having voice disorders, which could have influenced the sensitivity and specificity values. This underlines the importance of accounting for sociolinguistic variations, and voice registers when interpreting the discrimination accuracy of these assessments, emphasizing the need for ongoing investigation and refinement.

Furthermore, exploring the inclusion of bilingual speakers represents a valuable avenue for future research. Bilingual individuals may experience distinct vocal efforts and exhibit unique voice characteristics compared to monolingual speakers, adding depth to the intricate scenery of voice assessments [40,41]. Recognizing these multifaceted differences is pivotal for comprehensively evaluating voice quality using AVQI-3 and ABI. Incorporating concatenated samples, which enhance ecological validity by closely emulating real-world speaking situations, are uniquely poised to capture the nuanced variations in voice quality within bilingual contexts. Investigating the interplay between linguistic factors, vocal effort, and voice quality in bilingual speakers can significantly contribute to refining these tools and expanding their applicability across diverse linguistic settings.

In addition, while our research focused on US English speakers, acknowledging the global reach of English, with its numerous dialects, is crucial. Subsequent studies should examine the performance of acoustic indexes across various English-speaking populations with distinct accents and regional speech traits [42]. Future work involving more extensive settings and more comprehensive participant recruitment across multiple sites will enhance our understanding of these tools’ applicability and robustness in diverse linguistic and geographical contexts [43].

### 4.2. Clinical Utility

The ability of the AVQI-3 and ABI to differentiate the voice disorders group and the matched controls shows the value of multi-parameter indexes as a potential objective instrument for evaluating overall voice quality and breathiness in clinical and research settings. These tools could then contribute to identifying those with voice disorders, monitoring voice changes over time, and quantifying improvement from voice therapy interventions. As shown in other papers where the analysis of concatenated voice samples has shown therapeutic improvement [44], AVQI-3 and ABI significantly enhance ecological validity by closely mirroring real-world speaking situations [10,12,16]. This characteristic is significant in resource-constrained environments with limited access to advanced diagnostic tools. Furthermore, its robustness to background noise [45], a common challenge in various real-world settings such as healthcare and occupational environments, makes it particularly valuable [46]. However, further research is needed in these specific contexts.

Furthermore, the availability of AVQI-3 and ABI through freely accessible software such as Praat or VOXplot provides healthcare professionals with valuable tools for voice assessment. In practical terms, VOXplot simplifies calculating these voice quality indexes, making it a valuable resource for professionals in both research and clinical settings. Clinicians can effortlessly record and analyze the required voice samples for AVQI-3 and ABI calculations. Moreover, VOXplot allows for easy editing of audio files, enabling precise adjustments to the required SSN of 22 and a 3 s sustained vowel [a:]. This straightforward approach ensures accurate and reliable voice quality assessments while minimizing potential software-related challenges [47]. This accessibility empowers healthcare professionals to deliver comprehensive care and may stimulate the development of innovative voice therapies. 

While AVQI-3 and ABI validations do offer potential benefits in voice disorder management, it is important to note that further research on cost-effectiveness metrics for voice screening, assessment, and interventions is still warranted. 

Additionally, the value of a comprehensive voice assessment extends beyond these quantitative measures. In clinical practice, integrating AVQI-3 and ABI scores with auditory perceptual assessments and other standard practices is vital [1,2]. Auditory perceptual evaluation by experienced clinicians provides essential context, allowing for nuanced judgments that go beyond what is captured by numerical scores alone [22]. Combining objective tools with clinical expertise, this holistic approach ensures a more accurate and individualized voice quality assessment, enhancing the overall clinical management of voice disorders [33].

## 5. Conclusions

This current study assessed the utility of the Acoustic Voice Quality Index (AVQI-3) and the Acoustic Breathiness Index (ABI) in English speakers. The optimal thresholds for AVQI-3 and ABI achieved good discriminatory ability, with sensitivity ranging from 62% to 95% and specificity ranging from 81% to 84%, at thresholds of 1.17 and 2.35, respectively. Concurrent validity analysis revealed robust positive correlations between perceptual ratings and AVQI-3 as well as ABI (r_s_ = 0.72, *p* < 0.001; r_s_ = 0.77, *p* < 0.001), suggesting that AVQI-3 and ABI scores are significantly associated with auditory perceptual evaluations of overall voice quality and breathiness.

The findings suggest that AVQI-3 and ABI are promising tools for assessing overall voice quality and breathiness in US English speakers. Their robust discrimination accuracy and capacity to effectively identify and exclude positive and negative cases highlight their potential utility in clinical practice. The AVQI-3 helps identify hoarseness, while the ABI effectively recognizes breathiness in individuals. Both indexes could be used to monitor voice quality changes over time or evaluate the effectiveness of voice therapy interventions.

## Figures and Tables

**Table 1:** Age and gender distribution per voice diagnosis.

Diagnosis	Female		Male	
	Frequency	Mean Age (SD) in Years	Frequency	Mean Age (SD) in Years
Normal Voice	29	25.55 (5.58)	20	34.30 (12.77)
Atrophy	18	71.06 (11.92)	7	76.57 (6.60)
Bacterial Laryngitis	1	49	0	0
Carcinoma in Situ	0	0	2	64.00 (15.56)
Cyst	1	47	0	0
Erythema	1	50	1	57
Granuloma	2	58.50 (3.54)	1	35
Hemorrhage	1	49	0	0
Laryngeal Dystonia	7	56.00 (16.56)	3	74.00 (14.73)
Laryngeal Trauma	0	0	1	58
Laryngocele	1	80	0	0
Leukoplakia	2	50.00 (2.83)	4	54.50 (15.15)
Muscle Tension Dysphonia	7	42.14 (13.51)	5	43.40 (23.16)
Nodules	10	37.80 (17.24)	1	34
Papilloma	0	0	1	80
Paradoxical Vocal Fold Motion	3	67.67 (14.15)	0	0
Paresis	6	67.50 (10.59)	2	57.5 (17.68)
Paralysis	4	41.00 (18.78)	2	55.50 (14.95)
Posterior Glottic Diastasis	2	47.00 (1.41)	1	51
Reinke’s Edema	11	11.00 (57.91)	0	0
Scar	8	49.50 (45.67)	3	22.21 (17.62)
Supraglottic Mass	1	62	0	0
Tremor	3	65.33 (10.01)	1	74

**Table 2:** Comparison of Mean and Standard Deviation (SD) outcomes for Hand-Marked and Standardized Selection (SSN) of 22 Syllables in Continuous Speech (CS): Time, Acoustic Voice Quality Index (AVQI-3) and the Acoustic Breathiness Index (ABI) in US English.

Selection Method	Time (in Seconds)		AVQI-3		ABI	
	Mean	SD	Mean	SD	Mean	SD
Hand-marked	2.97272	0.26653	1.85	1.95	2.74	1.68
SSN (22 syllables)	2.96955	0.51102	1.82	1.95	2.72	1.69

## Data Availability

The data supporting the reported results in this paper are available from the corresponding author upon reasonable request.
